# A Complexed Crystal Structure of a Single-Stranded DNA-Binding Protein with Quercetin and the Structural Basis of Flavonol Inhibition Specificity

**DOI:** 10.3390/ijms23020588

**Published:** 2022-01-06

**Authors:** En-Shyh Lin, Ren-Hong Luo, Cheng-Yang Huang

**Affiliations:** 1 Department of Beauty Science, National Taichung University of Science and Technology, No. 193, Sec.1, San-Min Rd., Taichung City 403, Taiwan; eslin7620@gmail.com; 2 School of Biomedical Sciences, Chung Shan Medical University, No. 110, Sec.1, Chien-Kuo N. Rd., Taichung City 402, Taiwan; hong755225@gmail.com; 3 Department of Medical Research, Chung Shan Medical University Hospital, No. 110, Sec.1, Chien-Kuo N. Rd., Taichung City 402, Taiwan

**Keywords:** SSB, PriA, PriB, replication fork, primosome, OB-fold, myricetin, quercetin, flavonol, *Pseudomonas aeruginosa*

## Abstract

Single-stranded DNA (ssDNA)-binding protein (SSB) plays a crucial role in DNA replication, repair, and recombination as well as replication fork restarts. SSB is essential for cell survival and, thus, is an attractive target for potential antipathogen chemotherapy. Whether naturally occurring products can inhibit SSB remains unknown. In this study, the effect of the flavonols myricetin, quercetin, kaempferol, and galangin on the inhibition of *Pseudomonas aeruginosa* SSB (PaSSB) was investigated. Furthermore, SSB was identified as a novel quercetin-binding protein. Through an electrophoretic mobility shift analysis, myricetin could inhibit the ssDNA binding activity of PaSSB with an IC_50_ of 2.8 ± 0.4 μM. The effect of quercetin, kaempferol, and galangin was insignificant. To elucidate the flavonol inhibition specificity, the crystal structure of PaSSB complexed with the non-inhibitor quercetin was solved using the molecular replacement method at a resolution of 2.3 Å (PDB entry 7VUM) and compared with a structure with the inhibitor myricetin (PDB entry 5YUN). Although myricetin and quercetin bound PaSSB at a similar site, their binding poses were different. Compared with myricetin, the aromatic ring of quercetin shifted by a distance of 4.9 Å and an angle of 31° for hydrogen bonding to the side chain of Asn108 in PaSSB. In addition, myricetin occupied and interacted with the ssDNA binding sites Lys7 and Glu80 in PaSSB whereas quercetin did not. This result might explain why myricetin could, but quercetin could not, strongly inhibit PaSSB. This molecular evidence reveals the flavonol inhibition specificity and also extends the interactomes of the natural anticancer products myricetin and quercetin to include the OB-fold protein SSB.

## 1. Introduction

Single-stranded DNA (ssDNA)-binding protein (SSB) plays a crucial role in DNA replication, repair, and recombination as well as replication fork restarts [1]. SSB binds to ssDNA with a high affinity regardless of the sequence and prevents premature annealing, chemical attacks, and unwanted nuclease digestion [2]. Bacterial SSBs typically recognize ssDNA [3,4,5,6] via a highly conserved oligonucleotide/oligosaccharide-binding (OB) fold formed from a five-stranded β-barrel capped by an α-helix [7,8]. The eukaryotic counterpart of SSB is replication protein A (RPA). Although bacterial SSB [9] and RPA [10] share basic mechanistic functioning, they are different in terms of structure and many other functions [11,12,13,14,15,16]. Given the significant differences between RPA and SSB, the pharmacological inhibition of bacterial SSB may be used to target pathogens. The knowledge of the structure and of how bacterial SSB can be inhibited is an advantage for the development of inhibitors.

Cases of antibiotic-resistant bacterial infections are increasing at an alarming rate [17]. Few therapies are effective against the following six antibiotic-resistant pathogens: *Enterococcus faecium*, *Staphylococcus aureus*, *Klebsiella pneumonia*, *Acinetobacter baumannii*, *Pseudomonas aeruginosa*, and *Enterobacter* species (ESKAPE). These multidrug-resistant pathogenic bacteria are spreading rapidly worldwide and may become untreatable. Therefore, it is worth developing clinically useful small-molecule antibiotics to fight the growing threat of drug-resistant bacteria. Given that SSB is essential for all DNA-dependent cellular processes, SSB should be a prime target for antibiotic development.

The increased prevalence of beta-lactamases in *P. aeruginosa* and other bacteria has begun to reduce the clinical efficacy of beta-lactams against the most common opportunistic pathogens [18]. *P. aeruginosa* is a major opportunistic human pathogen that is responsible for nosocomial infections and infections in patients with impaired immune systems. *P. aeruginosa* has a remarkable capacity to develop a resistance to multiple classes of antimicrobial agents [19]. To date, over 800 beta-lactamases have been identified, of which at least 120 beta-lactamases have been detected in *P. aeruginosa* [20]. Thus, identifying new targets in microorganisms—that is, not focusing only on the bacterial cell wall synthesis proteins as targets—is a seminal event in the field of infectious diseases.

Phenolic compounds are a main class of secondary metabolites in plants and are divided into phenolic acids and polyphenols [21,22,23]. The antioxidant activity of phenolic compounds is attributed to the capacity of scavenging free radicals, donating hydrogen atoms, electrons, or chelate metal cations. Furthermore, many polyphenols [24,25] can be developed as drug candidates from the active confirmation of in vitro screens or in vivo evaluations [26]. These compounds have diverse physiological and pharmacological activities such as estrogenic, antitumor, antimicrobial, anti-allergic, and anti-inflammatory effects. Flavonoids are a family of polyphenolic compounds that are widespread in nature and are consumed as part of the human diet in significant amounts. Over 5000 different flavonoids have been identified, many of which display structure-dependent biological and pharmacological activities [27,28,29] including antimicrobial agents [30,31]. Flavonols belong to flavonoids, the most common group of plant polyphenols that are responsible for most of the flavor of fruit and vegetables [32]. Despite their broad antimicrobial activities [33], whether certain flavonols can inhibit bacterial SSB is still to be elucidated.

Phenolic compounds can interact with different proteins and may induce many effects upon binding [34,35,36]. Intermolecular binding is dominated by the stacking of polyphenolic rings onto planar hydrophobic surfaces and is strengthened by the multiple cooperative binding of polyphenolic rings [36]. Multiple interactions between polyphenols and protein may result in complexation, oligomerization, and precipitation [37]. The binding of phenolic compounds can also cause significant changes in the tertiary structure of protein [34], inhibit the enzyme activity, and influence the kinetic parameters [38]. Prior to this study, the binding effect of phenolic compounds on SSB was unknown.

**Figure 1:**
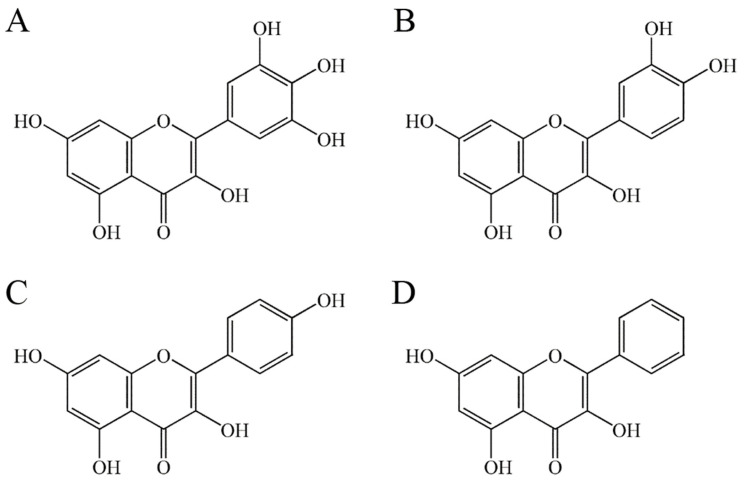
Molecular structure of (**A**) myricetin, (**B**) quercetin, (**C**) kaempferol, and (**D**) galangin.

In this study, we investigated the effects of the flavonols myricetin, quercetin, kaempferol, and galangin (Figure 1) on the inhibition of the binding activity of SSB from *P. aeruginosa* (PaSSB). These flavonols are composed of two aromatic rings linked by a heterocyclic pyran-4-one ring. For the first time, the flavonol myricetin was identified as an inhibitor of SSB with an IC_50_ of 2.8 ± 0.4 μM. The complexed crystal structure of PaSSB with the non-inhibitor quercetin (PDB entry 7VUM) was also determined to demonstrate the flavonol inhibition specificity of PaSSB.

## 2. Results

### 2.1. ssDNA Binding of PaSSB

**Figure 2:**
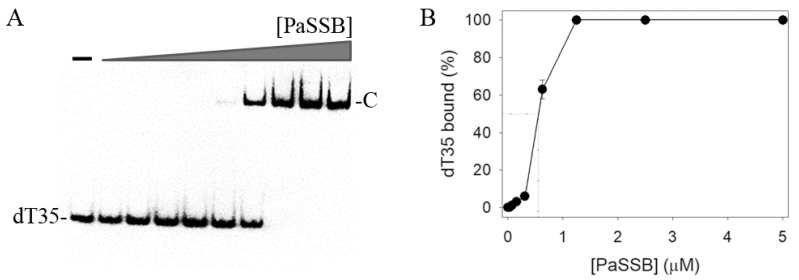
EMSA of PaSSB. (**A**) Purified PaSSB (0, 19, 37, 77, 155, 310, 630, 1250, 2500, and 5000 nM) was incubated with biotin-labeled dT35 at 37 °C for 60 min. (**B**) The titration curve. The [Protein]_50_ value of PaSSB was determined using an EMSA.

An electrophoretic mobility shift assay (EMSA) was used to analyze the ssDNA binding properties of PaSSB. The ssDNA dT35 was biotinylated at the 3′ terminal and incubated with purified PaSSB of different concentrations. The biotin-labeled dT35 could be detected by a streptavidin-horseradish peroxidase conjugate. As shown in Figure 2A, 1.25 μM PaSSB was sufficient to reach 100% binding of the dT35 ssDNA. Through the titration curve (Figure 2B), the binding constant of PaSSB was calculated to be 548 ± 36 nM.

### 2.2. Inhibition of the ssDNA Binding Activities of SSB by the Flavonol Myricetin

**Figure 3:**
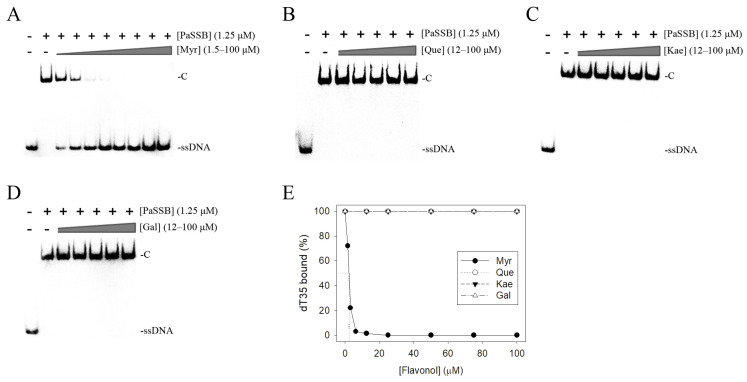
Inhibition of the ssDNA binding activity of PaSSB by flavonols. PaSSB (1.25 μM) was incubated with (**A**) myricetin, (**B**) quercetin, (**C**) kaempferol, and (**D**) galangin. These flavonols were dissolved in 10% dimethyl sulfoxide (DMSO). Quercetin, kaempferol, and galangin did not inhibit the binding of PaSSB to dT35. (**E**) An IC_50_ determination for PaSSB. PaSSB (1.25 μM) was incubated with myricetin (0, 1.5, 3.1, 6.3, 12.5, 25, 50, 75, and 100 μM), quercetin (12–100 μM), kaempferol (12–100 μM), and galangin (12–100 μM). Myricetin could inhibit the ssDNA binding activity of PaSSB with an IC_50_ of 2.8 ± 0.4 μM.

Although a complexed crystal structure of PaSSB with the flavonol myricetin has previously revealed where the binding occurs and the binding mode investigated [39], whether myricetin is an inhibitor of SSB was still undetermined. To assess whether myricetin inhibited the binding activity of SSB, myricetin (1.5–100 μM) was included in the binding assay (Figure 3A). A quantity of 1.25 μM PaSSB, i.e., a concentration sufficient to reach 100% binding of the dT35 ssDNA (Figure 2A), was used for this inhibition analysis. We found that myricetin could significantly inhibit PaSSB binding to dT35 (Figure 3A). According to the titration curve, the IC_50_ value of PaSSB for myricetin—that is, the inhibitor concentration required to reduce the binding activity of the protein by 50%—was 2.8 ± 0.4 μM.

### 2.3. The Flavonols Quercetin, Kaempferol, and Galangin Did Not Inhibit PaSSB

Myricetin, for the first time, was identified as a potent inhibitor of PaSSB. To clarify the flavonol inhibition specificity, quercetin, kaempferol, and galangin bearing different numbers of hydroxyl substituents on the aromatic rings (Figure 1)—namely, myricetin analogues, which may also inhibit the ssDNA binding activity of PaSSB—were used for the inhibition analysis (Figure 3B–E). Each of these flavonols (12–100 μM) was included in the binding assay. Unexpectedly, however, even at a concentration of 100 μM, quercetin, kaempferol, and galangin did not inhibit the binding of PaSSB to dT35. Thus, it was worth comparing the binding modes of these flavonols with PaSSB.

### 2.4. Crystal Structure of PaSSB in a Complex with Quercetin

**Figure 4:**
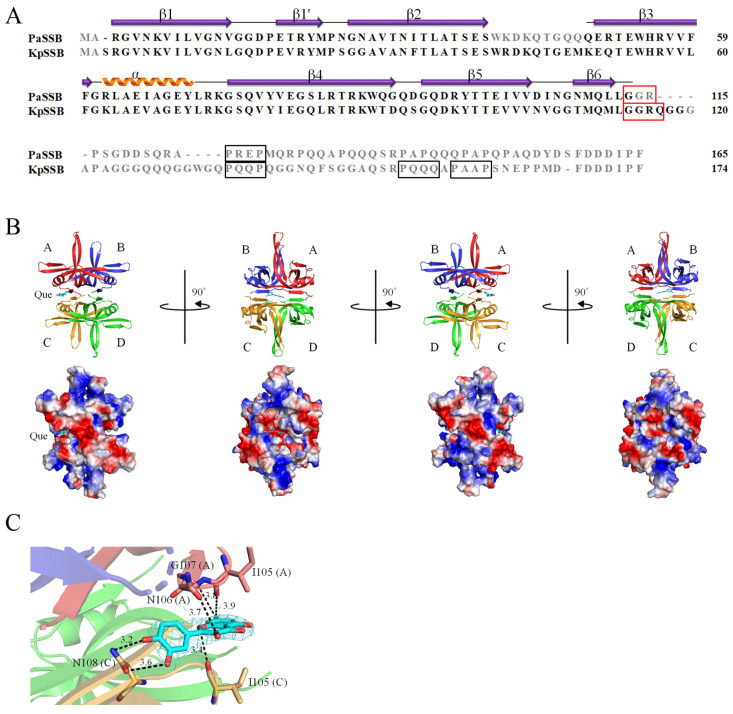
Crystal structure of PaSSB complexed with quercetin. (**A**) Sequence alignment of PaSSB and KpSSB. The corresponding PXXP motifs are boxed in black. The putative GGRQ motif is boxed in red. The secondary structural elements of KpPriB and KpSSB are shown with the sequences. The amino acids 114–165 (in gray) in the structure of PaSSB were not observed. (**B**) Complexed crystal structure of PaSSB with quercetin (PDB entry 7VUM). Four monomers of PaSSB were found per asymmetric unit. Even when complexed with quercetin, the amino acids 114–165 were not observed in the structure of PaSSB. A quercetin molecule could be found in a cavity created at the interface of PaSSB monomers A and C. (**C**) The interactions of PaSSB with quercetin. The electron density of quercetin was well-defined and indicated the presence of quercetin in the structure of PaSSB. Only one quercetin molecule was found in the structure of the PaSSB tetramer. Residues Ile105 (monomers A and C), Asn106 (monomer A), Gly107 (monomer A), and Asn108 (monomer C) of PaSSB were involved in quercetin binding.

Compared with myricetin, which bears three hydroxyl substituents, quercetin bears only two hydroxyl substituents on the aromatic ring (Figure 1). Myricetin was a potent inhibitor of PaSSB (Figure 3A) whereas quercetin was not (Figure 3B). Accordingly, establishing the precise differences in the binding modes of PaSSB between myricetin and quercetin was of considerable interest. Thus, we attempted to obtain a complexed structure of PaSSB with quercetin for a comparison. Crystals of PaSSB complexed with quercetin were grown at room temperature by hanging drop vapor diffusion in 25% PEG 4000, 200 mM magnesium chloride, 100 mM MES, and 100 μM quercetin at a pH of 6.5. The crystals of the quercetin-PaSSB complex belonged to space group P3_1_ with cell dimensions of a = 60.2, b = 60.2, and c = 131.4 Å. The complexed crystal structure of PaSSB with quercetin was determined at a 2.3 Å resolution (Table 1). Even when complexed with quercetin, the amino acids 114–165 were not observed in the structure of PaSSB (Figure 4A), suggesting that the C-terminal region in PaSSB was dynamic, which is similar to the case in *Escherichia coli* SSB (EcSSB) [13]. Four monomers of PaSSB were found in the asymmetric unit (Figure 4B). Consistently, PaSSB functioned as a tetramer [40].

The electron density of quercetin was well-defined and indicated the presence of quercetin in the structure of PaSSB (Figure 4C). A quercetin molecule could be found in a cavity created at the interface of PaSSB monomers A and C (Figure 4B). However, unlike the complexed structure of PaSSB with myricetin [39], which contained two myricetin molecules per tetramer, only one quercetin molecule was found in the structure of the PaSSB tetramer. Having a similar binding site to myricetin, but with a different binding pose, the quercetin molecule was sandwiched between PaSSB monomers A and C. The binding of quercetin did not influence the overall structure of PaSSB. Similar to the apo form [11], the global architecture of the quercetin-complexed PaSSB monomer revealed an OB-fold structure; i.e., a β-barrel capped with an α-helix.

Recently, we solved the crystal structure of SSB from *Klebsiella pneumonia* (KpSSB) [41]. The crystal structure of KpSSB revealed a GGRQ motif, which might be involved in regulating ssDNA binding. Based on the sequence alignment, the corresponding GGRQ motif in PaSSB was shortened to a GGR motif. Probably due to its flexibility, this motif was unobserved in our complexed structure of PaSSB (Figure 4A).

### 2.5. Quercetin Binding Mode

**Figure 5:**
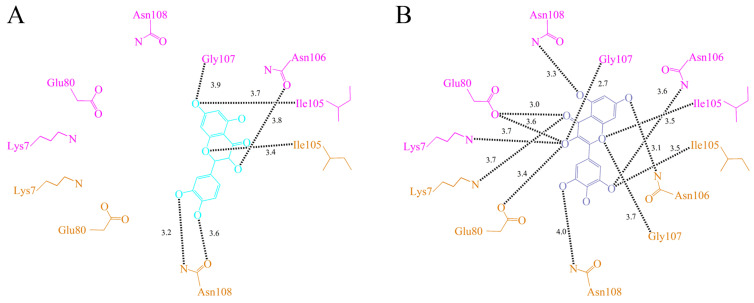
The binding modes. (**A**) Quercetin-binding mode of PaSSB. Residues Ile105, Asn106, Gly107, and Asn108 were involved in quercetin binding (PDB entry 7VUM). Monomers A and C of PaSSB are colored in pink and bright orange, respectively. (**B**) Myricetin-binding mode of PaSSB. Residues Lys7, Glu80, Ile105, Asn106, Gly107, and Asn108 in PaSSB were involved in myricetin binding (PDB entry 5YUN). For comparison, Lys7 and Glu80 in both monomers A and C of PaSSB interacted only with myricetin and not quercetin.

In this study, we found that SSB was a novel quercetin-interacting protein (Figure 4). Quercetin [32,42,43,44,45] has a wide range of biological activities and pharmaceutical relevance in anticancer, diabetes, aging, antioxidant, allergy, angioprotection, anti-inflammatory, anti-obesity, arthritis, asthma, exercise performance, gastroprotection, human prostate adenocarcinoma, immunity and infection, and mood disorder treatments. For the first time, quercetin was identified to be capable of binding to an OB-fold protein. As shown in Figure 5A, various interactions between quercetin and PaSSB were examined. Residues Ile105 (monomers A and C), Asn106 (monomer A), Gly107 (monomer A), and Asn108 (monomer C) within contact distance (<4 Å) were involved in quercetin binding. Gly107 and Asn108 formed hydrogen bonds with quercetin. We noticed that these interactions between quercetin and PaSSB were, however, significantly fewer than those for myricetin (Figure 5B). Residues Lys7 (monomers A and C), Glu80 (monomers A and C), Ile105 (monomers A and C), Asn106 (monomers A and C), Gly107 (monomers A and C), and Asn108 (monomers A and C) in PaSSB were involved in myricetin binding [39]. For comparison, Lys7 and Glu80 in both monomers A and C of PaSSB interacted only with myricetin and not quercetin.

### 2.6. The Flavonol Inhibition Specificity

**Figure 6:**
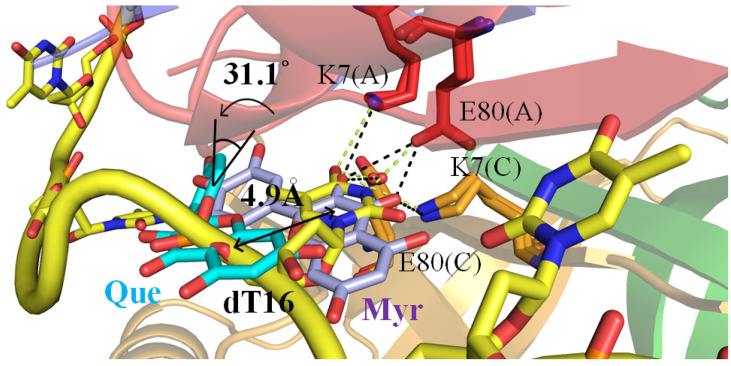
Structural comparison of the PaSSB complex among the quercetin, myricetin, and ssDNA-bound states of PaSSB. Monomers A and C of PaSSB are colored in red and bright orange, respectively. ssDNA is in gold. Quercetin is in cyan. Myricetin is in light blue. Superimposing these complexed structures revealed that both Lys7 and Glu80 (monomers A and C), which interacted with myricetin but not quercetin, were also crucial ssDNA binding sites. The side chain position of Lys7 and Glu80 was too distant to interact with quercetin. Compared with myricetin, the aromatic ring of quercetin shifted by a distance of 4.9 Å and an angle of 31° for hydrogen bonding to the side chain of Asn108 in PaSSB. Myricetin might compete with ssDNA for binding sites Lys7 and Glu80 and/or occupy these binding sites, thus preventing ssDNA from fully wrapping in PaSSB. These differences might be the reason why myricetin could, but quercetin could not, inhibit the ssDNA binding activity of PaSSB.

Our molecular evidence revealed that only a few residues in PaSSB—namely, Ile105, Asn106, Gly107, and Asn108—interacted with the non-inhibitor quercetin. Additional to these residues, Lys7 and Glu80 contributed to binding the inhibitor myricetin (Figure 5B). Thus, we checked whether Lys7 and Glu80 in PaSSB also interacted with ssDNA. Compared with the crystal structure of the PaSSB-ssDNA complex (PDB entry 6IRQ), both Lys7 and Glu80 are also known as crucial DNA binding sites [4]. Lys7 and Glu80 from monomers A and C may function similarly as a clamp to fix ssDNA binding (Figure 6). Myricetin might compete with ssDNA for binding sites Lys7 (monomers A and C) and Glu80 (monomers A and C) and/or occupy these binding sites, thus preventing ssDNA from fully wrapping in PaSSB. In addition, compared with myricetin, the aromatic ring of quercetin shifted by a distance of 4.9 Å and an angle of 31° for hydrogen bonding to the side chain of Asn108 in PaSSB (Figure 6). These differences might be the reason why myricetin could, but quercetin could not, inhibit the ssDNA binding activity of PaSSB.

## 3. Discussion

The development of clinically useful small-molecule drugs and the identification of new key targets have been seminal events in the field of antipathogen chemotherapies. DNA metabolism, such as the processes mediated by SSB, is one of the most basic biological functions and should be a prime target in antibiotic development. In this study, we found the flavonol myricetin, but not quercetin, was a potent inhibitor of PaSSB (Figure 3). Flavonols are safe as pharmaceuticals because they have few side effects for human use [32]. It is well-established that flavonols have several hydroxyl groups and, thus, have a marked potential for binding proteins. Despite the structures of myricetin and quercetin being similar, their binding modes to PaSSB were different (Figure 5).

Myricetin shows various pharmacological activities including antioxidant, anti-amyloidogenic, antibacterial, antiviral, antidiabetic, anticancer, anti-inflammatory, anti-epileptic, and anti-ulcer activities [46]. Myricetin is characterized by a pyrogallol B ring and the more hydroxylated structure is known to have increased biological properties compared with other flavonols [47]. Compared with myricetin, which has three hydroxyl groups, quercetin has only two hydroxyl groups on the B ring (Figure 1). Based on our crystal structure, we found that in the absence of one OH group, compared with myricetin, quercetin could not interact with Lys7 and Glu80. Both Lys7 and Glu80 are crucial DNA binding sites in PaSSB [4]. Thus, quercetin did not inhibit the ssDNA binding activity of PaSSB (Figure 6). In addition, we could not obtain complexed crystals with kaempferol and galangin for structural determinations. Compared with quercetin, either kaempferol or galangin had fewer hydroxyl groups on the B ring for hydrogen bonding to SSB. It is possible that they interacted with SSB with a weak affinity.

Many bacteria have more than one paralogous SSB [48] such as SsbA [12], SsbB [49,50], and SsbC [51] in *S. aureus* (Sa). In *E. coli*, PriB has also been identified as a type of SSB [52,53]. Whether these SSBs can be inhibited by myricetin is still to be elucidated. The important binding sites for myricetin in PaSSB—namely, Lys7 and Glu80 (Figure 5)—are Arg4 and Asp74 in SaSsbA [12], Arg4 and Asp74 in SaSsbB [50], Lys4 and Thr73 in SaSsbC [51], and Arg4 and Arg75 in KpPriB [54]. Based on these amino acid residue alignments, SaSsbA and SaSsbB have similar binding sites and are thought to be inhibited by myricetin. However, this speculation must be further biochemically and structurally investigated.

Myricetin could inhibit the ssDNA binding activity of PaSSB with an IC_50_ of 2.8 μM (Figure 3). The inhibitory effect of myricetin has been established in several helicases such as the SARS coronavirus helicase [55,56], the replicative DnaB helicase [57,58,59], the RSF1010 RepA helicase [60], and the PriA helicase [61]. Moreover, myricetin could also inhibit the activities of several cyclic amidohydrolases [62] such as the bacterial enzymes dihydropyrimidinase [63,64], dihydroorotase [65,66,67,68,69], and allantoinase [68,70]. Thus, myricetin may be a competent “dirty drug” (a multitarget drug) against ESKAPE pathogens [17] and has broad application prospects. The metabolic effects and safety of myricetin are well-established, thus making it beneficial for humans and as potential antibiotics for further development.

In conclusion, we analyzed the effects of the flavonols myricetin, quercetin, kaempferol, and galangin on the ssDNA binding ability of PaSSB. For the first time, our results demonstrated that a naturally occurring product, i.e., myricetin, was capable of inhibiting PaSSB activity. The complexed structures further revealed the flavonol inhibition specificity and extended the anticancer natural products of myricetin and quercetin interactomes to include the OB-fold protein, SSB. More complex structures of SSB with small molecules are useful to improve our understanding of how the bacterial primosome assembly [1,71,72,73,74,75] mediated by SSB can be inhibited.

## 4. Materials and Methods

### 4.1. Materials

All restriction enzymes and DNA-modifying enzymes were purchased from New England Biolabs (Ipswich, MA, USA) unless explicitly stated otherwise. All chemicals were purchased from Sigma-Aldrich (St. Louis, MO, USA) unless explicitly stated otherwise.

### 4.2. Protein Expression and Purification

The construction of the PaSSB expression plasmid has been previously reported [76]. The recombinant PaSSB protein was purified using the protocol described previously [77]. Briefly, *E. coli* BL21(DE3) cells were transformed with an expression vector and the overexpression of the expression plasmids was induced by incubation with 1 mM isopropyl thiogalactopyranoside. The protein was purified from the soluble supernatant by Ni^2+^ affinity chromatography (HiTrap HP; GE Healthcare Bio-Sciences, Philadelphia, PA, USA), eluted with Buffer A (20 mM Tris-HCl, 250 mM imidazole, and 0.5 M NaCl, pH 7.9), and dialyzed against a dialysis buffer (20 mM HEPES and 100 mM NaCl, pH 7.0; Buffer B). The protein purity remained at >97% as determined by SDS-PAGE (Mini-PROTEAN Tetra System; Bio-Rad, Hercules, CA, USA).

### 4.3. Crystallography

Purified PaSSB was concentrated to 18 mg/mL for crystallization. The crystals were grown at room temperature by hanging drop vapor diffusion in 25% PEG 4000, 200 mM magnesium chloride, 100 mM MES, and 100 μM quercetin at a pH of 6.5. The crystals reached the full size in 12–16 days. Data were collected using an ADSC Quantum-315r CCD area detector at an SPXF beamline BL13C1 at NSRRC (Taiwan). All data integrations and scaling were carried out using an HKL-2000 [78]. There were four PaSSB monomers per asymmetric unit. The crystal structure of PaSSB was solved at a 2.32 Å resolution with the molecular replacement software Phaser-MR [79] using PaSSB as the model (PDB entry 5YUO) [11]. A model was built and refined with PHENIX [80] and Coot [81]. The final structure was refined to an *R*-factor of 0.196 and an *R*_free_ of 0.250 (Table 1). The atomic coordinates and related structure factors were deposited in the PDB with the accession code 7VUM.

### 4.4. Labeling of the DNA Probe for EMSA

The 5′-biotinylated oligonucleotide (dT35) was synthesized for the EMSA. The final concentration of the labeled oligonucleotide was 30 fmol/μL.

### 4.5. EMSA

The EMSA [82] was conducted in accordance with a previously described protocol [83,84]. The EMSA was performed using a LightShift Chemiluminescent EMSA Kit (Thermo Scientific, Waltham, MA, USA) with a minor modification for PaSSB. In brief, PaSSB (0–5 μM) was incubated for 60 m at 37 °C with a DNA substrate (30 fmol/μL) in a total volume of 6 μL in 40 mM Tris-HCl (pH 7.5) and 50 mM NaCl. Following incubation, 4 μL of a dye mixture (0.01% bromophenol blue and 40% glycerol) was added. Native polyacrylamide gel (8%) was pre-electrophoresed at 110 V for 10 min. Thereafter, the resulting samples were loaded and resolved on pre-run gel and electrophoresed at 100 V for 1 h in a TBE running buffer (89 mM Tris borate and 1 mM EDTA). The protein-DNA complexes were electroblotted to a positively charged nylon membrane (GE, USA) at 100 V for 30 min in a fresh and cold TBE buffer. The transferred DNA was cross-linked with a nylon membrane using a UV light cross-linker instrument equipped with 312 nm bulbs for a 10 min exposure. Biotin-labeled DNA was detected using a streptavidin-horseradish peroxidase conjugate and a chemiluminescent substrate contained in a SuperSignal™ West Atto Ultimate Sensitivity Substrate (Pierce Biotechnology, Rockford, IL, USA). The ssDNA binding ability of the protein was estimated through linear interpolation from the concentration of the protein that bound 50% of the input DNA. To assess whether the flavonol inhibited the binding activity of SSB, PaSSB (1.25 μM) with a DNA substrate was individually incubated with myricetin (0, 1.5, 3.1, 6.3, 12.5, 25, 50, 75, and 100 μM), quercetin (12–100 μM), kaempferol (12–100 μM), or galangin (12–100 μM) for 60 m at 37 °C. The resultant protein solution was then analyzed using the EMSA.

## Figures and Tables

**Table 1:** Data collection and refinement statistics.

Data collection	
Crystal	PaSSB-quercetin
Wavelength (Å)	0.975
Resolution (Å)	30–2.32
Space group	P3_1_
Cell dimension (Å)	*a* = 60.2, *α* = 90°
	*b* = 60.2, *β* = 90°
	*c* = 131.4, *γ* = 120°
Completeness (%)	99.9 (99.9) *
<I/σI>	18.12 (2.44)
*R*_sym_ or *R*_merge_ (%)	0.064 (0.509)
Redundancy	3.2 (3.3)
Refinement	
Resolution (Å)	27.80–2.32
No. reflections	23090
*R*_work_/*R*_free_	0.196/0.250
No. atoms	
Protein	392
Water	64
R.m.s. deviation	
Bond lengths (Å)	0.008
Bond angles (°)	0.885
Ramachandran plot	
In preferred regions	360 (96.77%)
In allowed regions	12 (3.23%)
Outliers	0 (0%)
PDB entry	7VUM

* Values in parentheses are for the highest resolution shell. *R*_sym_ = Σ|I − ‘I’|/ΣI, where I is the observed intensity and ‘I’ is the statistically weighted average intensity of multiple observations of symmetry-related reflections.

## Data Availability

Atomic coordinates and related structure factors were deposited in the PDB with the accession code 7VUM.
